# Phase 1 study of MRX34, a liposomal miR-34a mimic, in patients with advanced solid tumours

**DOI:** 10.1038/s41416-020-0802-1

**Published:** 2020-04-02

**Authors:** David S. Hong, Yoon-Koo Kang, Mitesh Borad, Jasgit Sachdev, Samuel Ejadi, Ho Yeong Lim, Andrew J. Brenner, Keunchil Park, Jae-Lyun Lee, Tae-You Kim, Sangjoon Shin, Carlos R. Becerra, Gerald Falchook, Jay Stoudemire, Desiree Martin, Kevin Kelnar, Heidi Peltier, Vinicius Bonato, Andreas G. Bader, Susan Smith, Sinil Kim, Vincent O’Neill, Muhammad S. Beg

**Affiliations:** 1 The University of Texas MD Anderson Cancer Center, Houston, TX USA; 2 Asan Medical Center, Seoul, South Korea; 3 Mayo Clinic Cancer Center, Scottsdale, AZ USA; 4 Scottsdale Healthcare Research Institute, Scottsdale, AZ USA; 5 University of California Irvine Medical Center, Orange, CA USA; 6 Samsung Medical Center, Seoul, Korea; 7 The University of Texas Health Science Center at San Antonio, San Antonio, TX USA; 8 Seoul National University Hospital, Seoul, South Korea; 9 Severance Hospital, Seoul, South Korea; 10 Texas Oncology-US Oncology-Baylor University Medical Center, Dallas, TX USA; 11 Sarah Cannon Research Institute at HealthONE, Denver, CO USA; 12 Mirna Therapeutics, Austin, TX USA; 13 The University of Texas Southwestern Medical Center, Dallas, TX USA

**Keywords:** Drug development, Targeted therapies

## Abstract

**Background:**

In this first-in-human, Phase 1 study of a microRNA-based cancer therapy, the recommended Phase 2 dose (RP2D) of MRX34, a liposomal mimic of microRNA-34a (miR-34a), was determined and evaluated in patients with advanced solid tumours.

**Methods:**

Adults with various solid tumours refractory to standard treatments were enrolled in 3 + 3 dose-escalation cohorts and, following RP2D determination, expansion cohorts. MRX34, with oral dexamethasone premedication, was given intravenously daily for 5 days in 3-week cycles.

**Results:**

Common all-cause adverse events observed in 85 patients enrolled included fever (% all grade/G3: 72/4), chills (53/14), fatigue (51/9), back/neck pain (36/5), nausea (36/1) and dyspnoea (25/4). The RP2D was 70 mg/m^2^ for hepatocellular carcinoma (HCC) and 93 mg/m^2^ for non-HCC cancers. Pharmacodynamic results showed delivery of miR-34a to tumours, and dose-dependent modulation of target gene expression in white blood cells. Three patients had PRs and 16 had SD lasting ≥4 cycles (median, 19 weeks, range, 11–55).

**Conclusion:**

MRX34 treatment with dexamethasone premedication demonstrated a manageable toxicity profile in most patients and some clinical activity. Although the trial was closed early due to serious immune-mediated AEs that resulted in four patient deaths, dose-dependent modulation of relevant target genes provides proof-of-concept for miRNA-based cancer therapy.

**Clinical trial registration:**

NCT01829971.

## Background

MicroRNAs (miRNAs) are naturally occurring non-coding RNA molecules, usually ~17–23 nucleotides (nt) long, which comprise a new class of ‘master regulators’ of gene expression.^1^ miRNAs act post-transcriptionally to simultaneously modulate the expression of up to several hundreds of genes and have a ubiquitous involvement in physiological and pathological processes in animals.^1,2^ Accordingly, dysregulation of miRNA expression itself is associated with human diseases such as cancer.^1–4^ miRNAs have the ability to function as conventional tumour suppressors or oncogenes^5,6^ and therefore have the potential to serve as new cancer therapy targets.^5–8^ The introduction of a miRNA mimic to restore the functionality of a tumour suppressor miRNA whose expression is lost or downregulated in the tumour is one therapeutic approach under careful consideration.^9–11^

MRX34 is a liposomal formulation of miR-34a and a potential first-in-class miRNA mimic cancer therapy.^12–14^ miR-34a is a naturally occurring tumour suppressor that is lost or expressed at reduced levels in a broad range of tumour types.^7,15–17^ Retrospective clinical studies have demonstrated a negative correlation of low miR-34 expression to survival in a number of different cancer types.^18^ In normal tissue, miR-34a is involved in the down-regulation of expression of over 30 unique oncogenes, including but not limited to MET, MYC, PDGFR-α, CDK4/6 and BCL2; genes involved in tumour immune evasion, such as PD-L1 and DGKζ, were also found to be regulated by miR-34a.^7,19–21^ Exogenous introduction of miR-34a mimics in vitro resulted in reduced cell proliferation, migration and invasion; synergistic effects were also observed when miR-34a mimics were combined with anti-cancer therapies.^7,8,22–26^ In pre-clinical animal models, miR-34a delivered by a variety of vehicles inhibited primary tumour growth, blocked metastasis, and improved survival.^7,12,24,25,27,28^ Moreover, orthotopic mouse models of hepatocellular carcinoma (HCC) displayed significant growth inhibition and tumour regression in more than a third of MRX34-treated animals.^12^

We previously reported initial Phase 1 results from adult patients with refractory advanced solid tumours treated with escalating twice-weekly (BIW) doses of MRX34.^14^ Here, we report the final results, including pharmacodynamics and determination and evaluation of the recommended Phase 2 dose (RP2D) of an alternate daily treatment schedule (QDx5) in expansion cohorts in this first-in-human clinical trial of a miRNA-based cancer therapy.

## Methods

### MRX34

As described previously, MRX34 is a synthetic, double-stranded miR-34a mimic that is 23-nt in length and encapsulated in a liposomal nanoparticle (Fig. S1).^12–14,29,30^

### Patients

Eligible patients were aged ≥18 years and had refractory solid tumours for which no standard treatment existed, ECOG performance status 0–2, acceptable hepatic, renal, and haematologic function, and anticipated life expectancy of at least 3 months. For patients with HCC, only those with liver disease classified as Child-Pugh A were eligible. While patients with asymptomatic CNS metastases were initially included, the protocol was amended to exclude them after CNS haemorrhages occurred in two patients. Likewise, after tumour shrinkage was observed in metastatic sites in non-HCC patients without hepatic metastases, the protocol was amended to include such patients. Expansion cohorts accepted patients with a variety of cancer types, including viral-related HCC, melanoma (non-cutaneous, excluding uveal), small cell lung cancer, triple-negative breast cancer, sarcoma, and bladder, renal, and ovarian cancers. The study followed the Declaration of Helsinki and the International Conference on Harmonization Good Clinical Practice guidelines. Patients were enrolled with approval from the ethics committees and institutional review boards at participating institutions, and all patients provided written informed consent before starting study-specific procedures.

### Study design

As in the initial stage of this multi-centre, open-label, dose escalation/expansion Phase 1 clinical trial in which patients received MRX34 twice-weekly for 3 weeks in a 4-week cycle (BIW) schedule,^14^ eligible patients in the United States and the Republic of Korea were enrolled into 3 + 3 dose-escalation cohorts. In this final part of the study, MRX34 was given intravenously daily for 5 days along with dexamethasone (DEX) pre-medication twice daily (BID) for 7 days in week 1, followed by 2 weeks of rest in 3-week cycles (QDx5 schedule). Based on immune-mediated toxicity including both infusion-related and later-occurring adverse events (AEs) observed with the BIW schedule,^14^ the protocol was amended to require DEX 10 mg BID pre-medication in the first cycle, which could then be reduced in subsequent cycles at the investigators’ discretion. Each dose of MRX34 was infused over 2–4 h with a controlled infusion pump without filtration. The MRX34 dose was escalated from 50 to 70, 93 and finally 110 mg/m^2^ using a modified Fibonacci scheme. Expansion cohorts were then enrolled at the RP2Ds identified, which were different for HCC and non-HCC tumours. In late May 2016, the protocol was again amended to allow an induction period of three cycles followed by an observation period with no treatment, with the intent of mitigating any DEX-related dampening of anti-tumour immune responses potentially triggered by MRX34. However, the study was closed before this schedule could be fully explored. The primary objective was to determine the RP2D; secondary objectives included assessments of safety and tolerability, pharmacokinetics (PK), pharmacodynamics, and clinical activity.

### Study evaluations

All patients treated with MRX34 were evaluated for safety, with adverse events (AEs) graded by NCI-CTCAE version 4.03 and attributed by the investigator. AEs and concomitant medications were recorded at each patient visit, with laboratory abnormalities recorded separately. A Cohort Review Committee comprised of the investigators, site coordinators, and medical monitor met weekly via teleconference to review safety and manage the dose-escalation process. An AE was considered a dose-limiting toxicity (DLT) if it occurred during cycle 1, was clinically significant, grade 3 or 4, and related to study treatment. Grade 3 nausea, vomiting, diarrhoea, or cytokine release syndrome (CRS) related to infusion reactions and associated with suboptimal prophylactic and other supportive treatment were not considered DLTs. Anti-tumour activity was evaluated by CT or MRI performed at screening, at the end of cycle 2, and then after every even cycle. If a response was noted (RECIST version 1.1), follow-up radiographic assessment was performed at ≥4 weeks (>28 days) for confirmation.

### Pharmacokinetics

Blood samples for PK analysis of MRX34 administered on the QDx5 schedule were collected in cycle 1 pre-infusion and at multiple timepoints post-infusion on days 1–5, and then once daily on days 6, 7, 8 and 15. Concentrations of miR-34a mimic extracted from blood samples were measured by a validated hybridisation-ELISA assay at Charles River Laboratories. PK parameters were estimated from blood concentration versus time profiles using commercial software (Phoenix WinNonLin, Pharsight) and a non-compartmental model.

### Pharmacodynamics

In planned pharmacodynamics studies, white blood cells (WBCs) were isolated (LeukoLOCK fractionation method, ThermoFisher Scientific, Waltham MA; Leukosorb filter, Pall, Port Washington, NY) from whole blood collected from patients at each dose level. WBC samples were collected pre-dose and at five different timepoints, up to 150 h, post-MRX34 dosing. RNA in WBCs was isolated (mirVana RNA isolation kit, ThermoFisher Scientific, Waltham, MA), and its quality assessed (Agilent 2100 Bioanalyzer, Agilent, Santa Clara, CA). RNA samples were then measured and compared for pre- and post-MRX34 expression of selected direct miR-34a target genes using both qRT-PCR and next generation sequencing (NGS; RNA-Seq). For the NGS assay, RNA characterisation and molecular profiling were performed via poly(A) selection, and RPKM (Reads Per Kilobase of transcript per Million mapped reads) was used to determine relative gene expression levels. To determine the global effect of MRX34 treatment on gene expression, we performed enrichment analysis by evaluating mRNAs carrying putative miRNA binding sites to be significantly enriched among transcripts downregulated 24 h post-MRX34 treatment, a process similar to the Sylamer or Sylarray algorithm.^31,32^ The data set for this analysis consisted of data from 70 samples across all cohorts (33–100 mg/m^2^), but was restricted to 14,000 mRNAs showing an average expression of >0.5 log-transformed RPKM. In addition, a chromogenic in situ hybridisation (CISH) staining method was developed to visualise cellular and sub-cellular localisation of miR-34a following MRX34 treatment, and was applied to pre- and post-treatment liver biopsies from patients with various advanced cancer types. Briefly, formalin-fixed and paraffin-embedded tissues on glass slides were deparaffinised with xylene, washed with 70% ethanol and phosphate-buffered saline, treated with proteinase K, and dehydrated in ethanol. Tissues were then incubated with a miR-34a specific capture probe (miRCURY LNA, Qiagen, cat. No. 38487-15) under a cover slip, rinsed with saline-sodium citrate buffer, labelled with anti-digoxigenin (Qiagen), and counterstained with Fast Red.

### Statistical analyses

All patients receiving at least one dose of MRX34 were included in the analyses of safety and response. Other than specialised methods developed for interpretation of large NGS data sets (e.g., Sylamer analysis), only descriptive statistics were used to evaluate results.

## Results

### Patients and drug exposure

From September 2014 to September 2016, we treated 85 patients with at least one dose of MRX34 using the QDx5 schedule. Many of the patients were Asian (49%), most were male (73%), and median age was 60 years (Table 1). Tumour types included HCC (*n* = 36), melanoma (*n* = 9), renal cell carcinoma (*n* = 8), lung cancer (*n* = 8), and a variety of other cancers. All patients had good performance status (0, 20%; 1, 80%), and all had been treated with prior therapies for advanced cancer (median 3 prior therapies, range, 1–11), with a third (*n* = 28) having received ≥4 prior therapies. Twenty-five (29%) patients received a single cycle (up to five doses) of MRX34, although eight of these received a single cycle due to early termination of the study, and a third of patients (*n* = 28) received ≥3 cycles. Forty-three (50%) patients discontinued the study due to disease progression, 14 (16%) withdrew consent for various reasons and six (7%) discontinued due to AEs.

**Table 1:** Patient demographics, disease characteristics, and MRX34 treatment exposure.

Characteristic	Patients (*N* = 85)
Age, median (range)	60 (32–81)
Sex: male, *n* (%)	62 (73)
Race: Asian/Caucasian/Black/Other, %	49/33/2/16
ECOG Performance Score 0/1, %	20/80
Cancer type, *n* (%)	
Hepatocellular carcinoma	36 (42)
Melanoma	9 (11)
Renal cell carcinoma	8 (9)
Lung^a^	8 (9)
Gastrointestinal stromal tumour	6 (7)
Neuroendocrine	6 (7)
Other	12 (14)
Prior therapies, median (range)	3 (1–11)
MRX34 cycles delivered, median (range)	2 (1–16)
MRX34 cycles delivered, *n* (%)	
1^b^	25 (29)
2	32 (38)
3–4	13 (15)
≥5	15 (18)

^a^Includes five small cell, two adenocarcinoma, one squamous.

^b^Includes eight patients who received only one cycle due to early termination of the study.

### Safety

Most AEs and laboratory abnormalities were grade 1 or 2, with many related to infusion reactions (i.e., fever, chills, back pain). Although grade 3/4 laboratory abnormalities were frequently recorded, most were not associated with clinical symptoms. The most frequently observed non-laboratory adverse events, regardless of grade or relationship to MRX34, were fever (percentage all grades/grade 3: 72/4), chills (53/14), fatigue (51/9), back/neck pain (36/5), nausea (36/1), and dyspnoea (25/4) (Table 2). Laboratory abnormalities included lymphocytopenia (percentage grade 3/grade 4: 44/18), thrombocytopenia (29/6), neutropenia (21/8), increased AST (11/2), increased ALT (11/1), hyperglycaemia (17/2) and hyponatremia ((17/2). Treatment-attributed serious AEs (SAEs) tended to occur late in the cycle (after completion of daily MRX34 infusions) and included sepsis, hypoxia, cytokine release syndrome, and hepatic failure, a pattern suggestive of immune-mediated toxicity. Following the first incidence of hepatitis, liver function eligibility criteria became more stringent along with more frequent monitoring of liver function. Patients with HCC were given allopurinol or its equivalent before the first dose of MRX34 and thereafter as clinically appropriate to prevent potential tumour lysis syndrome. The study was terminated after five drug-related SAEs occurred in the expansion cohorts, namely enterocolitis, hypoxia/systemic inflammatory response syndrome, colitis/pneumonitis, hepatic failure, and cytokine release syndrome/respiratory failure, the latter four of which resulted in death.

**Table 2:** Adverse events and laboratory abnormalities in patients with advanced solid tumours treated with daily MRX34 monotherapy.

	Overall (*N* = 85)			Dosing cohort, mg/m^2^								
				70 (*n* = 30)			93 (*n* = 42)			110 (*n* = 6)		
AEs in ≥20% of all patients, *n* (%)	All	G3	G4	All	G3	G4	All	G3	G4	All	G3	G4
Fever	61 (72)	3 (4)	–	22 (73)	–	–	29 (69)	–	–	5 (83)	–	–
Chills	45 (53)	12 (14)	–	18 (60)	–	–	19 (45)	–	–	4 (67)	–	–
Fatigue	43 (51)	8 (9)	–	13 (43)	1 (3)	–	24 (57)	5 (12)	–	3 (50)	1 (17)	–
Back/neck pain	31 (36)	4 (5)	–	12 (40)	1 (3)	–	13 (31)	2 (5)	–	3 (50)	–	–
Nausea	31 (36)	1 (1)	–	11 (37)	1 (3)	–	14 (33)	–	–	4 (67)	–	–
Abdominal pain	24 (28)	2 (2)	–	7 (23)	2 (7)	–	13 (31)	–	–	1 (17)	–	–
Dyspnoea	21 (25)	3 (4)	1 (1)	9 (30)	1 (3)	–	9 (21)	–	1 (2)	2 (33)	1 (17)	–
Decreased appetite	28 (33)	2 (2)	–	7 (23)	1 (3)	–	16 (38)	–	1 (2)	2 (33)	–	–
Headache	22 (26)	–	–	8 (27)	–	–	10 (24)	–	–	3 (50)	–	–
Serious AEs (SAEs)	48 (56)	20 (24)	9 (11)	17 (57)	10 (33)	2 (7)	25 (60)	9 (21)	6 (14)	–	–	–
Treatment-related SAEs	32 (38)	14 (16)	4 (5)	11 (37)	7 (23)	1 (3)	17 (40)	5 (12)	3 (7)	–	–	–
On-study deaths^a^	8 (9)			2 (7)			3 (7)			1 (17)		

Laboratory abnormalities	**G2**	**G3**	**G4**	**G2**	**G3**	**G4**	**G2**	**G3**	**G4**	**G2**	**G3**	**G4**
Lymphopenia	17 (20)	37 (44)	15 (18)	8 (27)	14 (47)	5 (17)	6 (14)	23 (55)	7 (17)	3 (17)	2 (33)	–
Thrombocytopenia	23 (27)	25 (29)	5 (6)	9 (30)	11 (37)	2 (7)	11 (26)	12 (26)	3 (7)	1 (17)	2 (33)	–
Neutropenia	19 (22)	18 (21)	7 (8)	8 (27)	7 (23)	3 (10)	10 (24)	9 (21)	3 (7)	1 (17)	–	1 (17)
Decreased albumin	31 (36)	7 (8)	–	9 (30)	3 (10)	–	18 (43)	3 (7)	–	2 (33)	1 (17)	–
Increased ALT	11 (13)	9 (11)	1 (1)	6 (20)	2 (7)	–	4 (10)	5 (12)	1 (2)	1 (17)	–	–
Increased AST	15 (18)	9 (11)	2 (2)	6 (20)	7 (23)	1 (3)	6 (14)	2 (5)	1 (2)	–	1 (17)	–
Increased bilirubin	3 (4)	5 (6)	–	3 (10)	2 (7)	–	–	3 (7)	–	–	–	–
Hyperglycaemia	36 (42)	14 (17)	2 (2)	12 (40)	4 (13)	1 (3)	19 (45)	8 (19)	1 (2)	4 (67)	1 (17)	–
Hyponatremia	–	14 (17)	2 (2)	–	7 (23)	–	–	4 (10)	2 (5)	–	2 (33)	–

^a^From initial dose to 30 days after last dose; possibly/probably/definitely related deaths included deaths from sepsis, hepatic failure, hypoxia, cytokine release syndrome; unlikely-to-be-related/not related deaths included deaths from progressive disease, pulmonary embolism, cardiac arrest, and pneumonia.

Study drug-related deaths are as follows: (1) the first patient was a 77-year-old renal cell carcinoma patient that initially presented with a 24-h history of bloody diarrhoea. The patient subsequently experienced worsening hypoxaemia, possible colitis, pulmonary failure, and marked disease progression in both lungs. Despite dexamethasone pre-medication, the subject may have experienced an immune-mediated adverse reaction in the lung and colon. (2) The second MRX34-related death involved a 73-year-old patient with metastatic small cell lung cancer who initially presented with dyspnoea, chest pain, generalised weakness and bloody stool. Disease progression in the liver was evident. The patient passed away secondary to multi-organ failure. (3) The third patient was a 35-year-old metastatic melanoma patient who initially presented with generalised weakness. A CT scan of the brain was performed following a seizure accompanied by disobedient mentality but did not show any evidence of bleeding. The patient’s clinical condition continued to rapidly decline and he subsequently expired. (4) Lastly, a 60-year-old hepatocellular carcinoma patient experienced bronchopulmonary haemorrhage, became increasingly hypoxic, and experienced a generalised seizure. The patient opted for hospice care following a delay in the recovery of neurologic status and expired soon after due to cytokine release syndrome.

### RP2D Determination

For patients with HCC, five of six at the 93 mg/m^2^ dose level could not receive the full five days of MRX34 in cycle 1 due to neutropenia, thrombocytopenia, and pneumonia. For patients with non-HCC tumours, four of six experienced DLTs at the 110 mg/m^2^ dose level, including hypoxia, thrombocytopenia, and cycle 2 delays due to neutropenia and thrombocytopenia. No DLTs were reported for either group at previous dose levels. Based on these results, the RP2D was determined to be 70 mg/m^2^ for patients with HCC and 93 mg/m^2^ for patients with non-HCC tumours.

### Pharmacokinetics

As with the BIW schedule,^14^ blood concentration versus time curves for the QDx5 schedule showed variability within and between dose levels (Fig. S2). Large standard deviations were similarly observed for all PK parameters (Table S1), but showed non-linear, non-dose proportional increases in Cmax, T_½_, and AUC with increasing MRX34 dose levels. At all dose levels, clearance slowed by day 5 such that AUC increased by approximately one log on day 5 compared with day 1. These effects are consistent with frequency- and dose-related saturation of the reticular endothelial system (RES), which is presumed to clear the MRX34 liposomes as observed in pre-clinical studies, potentially resulting in higher exposure of non-RES tissues, including tumours, to MRX34.

### Pharmacodynamics

**Fig. 1:**
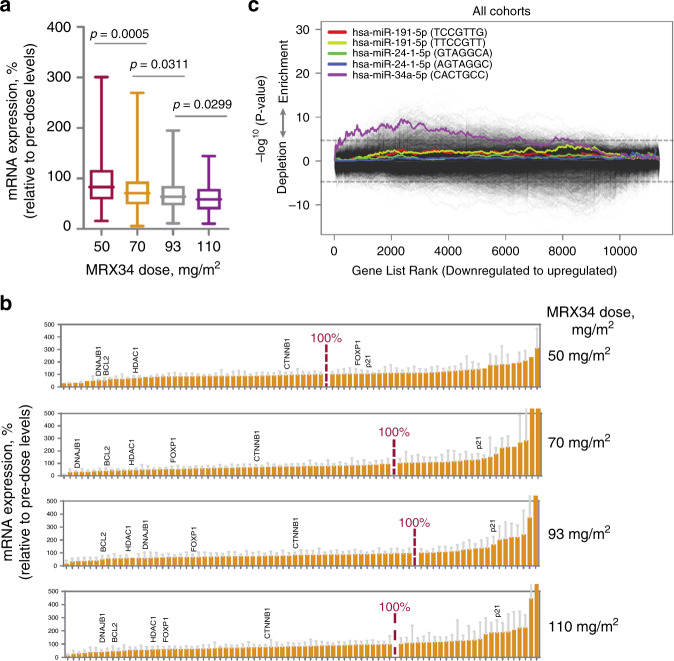
MRX34 pharmacodynamics. **a** As measured by qRT-PCR, combined relative mRNA expression (%) pre- and post-treatment (5 timepoints) of five miR-34a target oncogenes (BCL2, CTNNB1, DNAJB1, FOXP1, HDAC1) in white blood cells (WBCs) from patients shows dose-dependent downregulation with increasing MRX34 dose from 50 (*n* = 4) to 70 (*n* = 16) to 93 (*n* = 16) to 110 mg/m^2^ (*n* = 9). Average expression in pre-dose samples was set as 100%. **b** Next generation sequencing (RNA-Seq) measurement of relative mRNA expression (%) pre- and post-treatment (24 h) for validated miR-34a target genes shows similar results to qRT-PCR. Dose-dependent knockdown is suggested by the increased range of downregulated genes seen with increasing MRX34 dose from 50 (*n* = 4) to 70 (*n* = 6) to 93 (*n* = 9) to 110 mg/m^2^ (*n* = 11). Validated miR-34a target genes contain miR-34a binding sites in their respective 3′ UTRs for which regulation by miR-34a has been experimentally verified. **c** Sylamer analysis plot shows a statistically significant enriched miRNA signature for miR-34a for putative target genes, suggesting specific activity of the miR-34a mimic against its target genes in patient-derived WBCs. In this analysis, genes affected by MRX34 at the 24-hr time point relative to baseline in the pre-dose samples for all dosing cohorts were sorted based on RNA-Seq measured expression from most downregulated to most upregulated. The 3′ UTR sequences of these genes were then scanned by the Sylamer algorithm for miRNA binding sites. The *x*-axis of the plot represents the sorted gene list from downregulated to upregulated. The y-axis represents an enrichment score of the seed sequence binding sites. *P*-values are based on simulations for seed ‘CTGCCA’. The plot also includes results of Sylamer analyses performed for house-keeping miRNAs, miR-24 and miR-191, which failed to show significant enrichment of downregulated target genes for these miRNAs, further indicating specific gene-directed activity of MRX34.

**Fig. 2:**
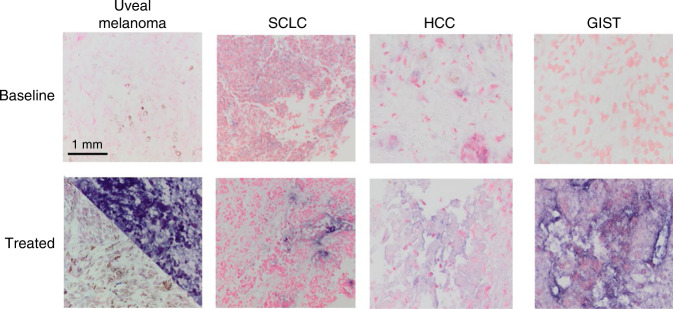
Chromogenic in situ hybridisation (CISH) staining of pre- (baseline) and post-MRX34 treatment (treated) liver biopsies from patients with various advanced solid tumours. Compared to pre-treatment staining, post-treatment results show variably increased miR-34a staining (dark blue/purple) in tumour tissue with localisation to the cellular cytoplasm. In all cases, a lesion in the liver was biopsied for CISH analysis. Uveal melanoma: tumour biopsy was taken 12 days after first dose (93 mg/m^2^); the biopsy presented spindle cell (bottom) and polygonal-shaped (top) melanoma. SCLC: tumour biopsy was taken 3 days after MRX34 dosing (93 mg/m^2^). HCC: tumour biopsy was taken 3 days after MRX34 dosing (70 mg/m^2^). GIST: tumour biopsy was taken 4 days after MRX34 dosing (93 mg/m^2^).

During pre-clinical evaluation of MRX34, we identified a 5-gene mRNA signature as a molecular pharmacodynamic readout in circulating WBCs for MRX34 bioactivity in vivo. These mRNAs, BCL2,^33–36^ DNAJB1,^37^ CTNNB1,^38^ FOXP1,^39,40^ and HDAC1,^41^ carry 3′ UTR sites that are targeted and repressed by miR-34. qRT-PCR analysis of RNA from WBCs in blood samples from patients showed down-regulation of miR-34a target genes that appears to be dose-dependent, and both greater and longer-lasting at higher MRX34 dose levels (Fig. 1a). In contrast, expression levels of p21 (CDKN1A), a tumour suppressor gene specifically induced by miR-34a,^41^ was increased in a dose-dependent manner (data not shown). These results were confirmed by NGS (RNA-Seq) analyses of the same WBC samples to quantify expression of 99 direct miR-34 target genes (Fig. 1b), and also by unbiased enrichment analysis of NGS data (Fig. 1c). CISH staining of pre- and post-treatment liver biopsies from patients with various tumour types showed increased miR-34a in tumour tissues following MRX34 treatment, localised to the cellular cytoplasm, verifying delivery of miR-34a to the tumour microenvironment (Fig. 2).

### Efficacy

Of the 66 patients who were evaluable for response, 16 had clinically significant SD for ≥4 cycles, with a median duration of 19 weeks (range, 11–55) (Table 3). There were no CRs, but PRs were confirmed for three patients (ORR, 4%). The remaining 31 patients had PD as best response.

**Table 3:** Best overall response in patients with advanced solid tumours treated with once daily MRX34 monotherapy.

Best overall response, *n* (%)	Patients **(** *N* = 85)
CR	0
PR^a^	3 (4)
SD	
≥1 cycle	32 (37)
≥4 cycles^b^	16 (19)
PD	31 (37)
Not evaluable	19 (54)

^a^Includes one patient each with acral melanoma, clear cell renal carcinoma, and HBV-related hepatocellular carcinoma. Durations of response for these patients were 65+, 54 and 12 weeks, respectively.

^b^Median duration 136 days (range, 79–386).

**Fig. 3:**
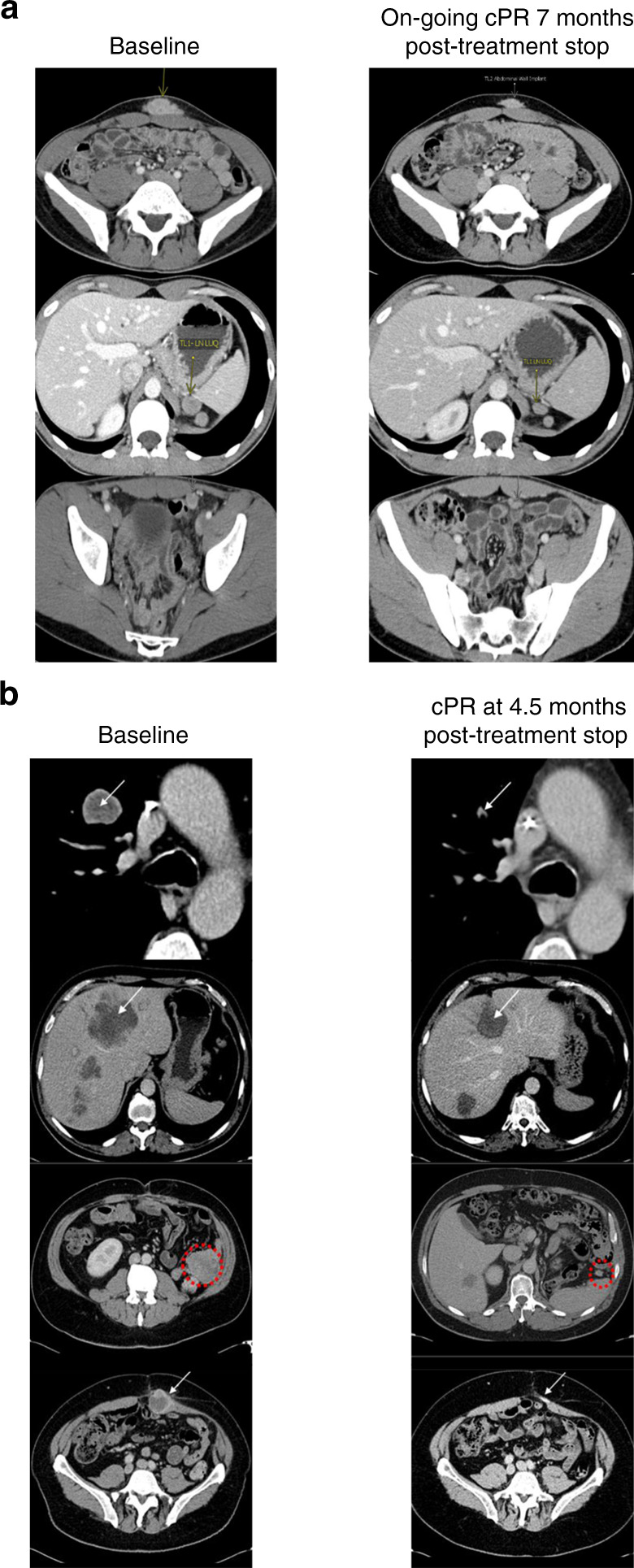
Responses in patients treated with MRX34. **a** Confirmed PR in a 32-year-old male with N-ras mutated, KIT/BRAF wild, PD-L1^+^ acral melanoma that was initially treated by thumb amputation. Following progression with multiple metastases, the patient was treated unsuccessfully with adoptive T-cell therapy with high-dose IL-2, ipilimumab, pembrolizumab, and CVT chemotherapy. MRX34 treatment was initiated and index lesion size reductions of 39 and 54% were observed after cycles 4 and 6, respectively. MRX34 was discontinued at the patient’s request after completion of cycle 7, after which the PR lasted an additional 7 months with no other treatment for a total duration of response of 65+ weeks. **b** Confirmed PR in a 56-year-old male with clear cell renal carcinoma who had been refractory to sunitinib, temsirolimus, and bevacizumab. The patient received three cycles of MRX34. Due to rising liver enzymes, treatment was discontinued at that time, and a liver biopsy showed immune hepatitis, but no tumour. PR was noted 3 months after MRX34 discontinuation and confirmed at 4.5 months. The response lasted 54 weeks.

All three responding patients had been heavily pre-treated, and their responses had characteristics, including appearance and maintenance after drug discontinuation, potential pseudo-progression in one case, and extended duration, suggestive of immune-mediated anti-tumour activity. The first responding patient, a 31-year-old man with acral melanoma, had previously received adoptive T-cell therapy with high-dose IL-2, ipilimumab, and pembrolizumab. PR was confirmed in cycle 5 and continued after MRX34 therapy was discontinued, with no other therapy given, lasting for 65+ weeks (Fig. 3a). In the second patient, a 56-year-old man with clear cell renal carcinoma, PR was reported 3 months (confirmed at 4.5 months) after MRX34 had been given for 3 cycles and discontinued due to rising liver enzymes (Fig. 3b). Liver biopsies at the time of MRX34 discontinuation showed necrotic tumour and immune hepatitis. The response lasted 54 weeks until PD was confirmed by central review, although the patient had continued to do well clinically without additional treatments when last seen in follow-up in October 2016. The third responder, a 33-year-old woman with HBV-related HCC, had received two cycles of MRX34 which was discontinued for PD. Her PR was revealed on a CT scan 2 months later and lasted for 12 weeks with no further treatment.

## Discussion

This first-in-human clinical trial of a miRNA-based therapy was closed early due to unexpectedly severe immune-mediated toxicities, which resulted in four patient deaths in expansion cohorts. While the QDx5 schedule proved more practical than the BIW schedule to deliver in the clinic, it was still associated with serious AEs and no apparent increase in activity. It was therefore concluded that the risk of serious immune-related AEs was not outweighed by the 4% overall response rate, and clinical development of MRX34 has been halted. Pharmacodynamic data did demonstrate dose-dependent modulation of miR-34a target genes in patients (albeit in WBCs and not in tumour) and miR-34a localisation in tumours, supporting our initial rationale for pursuing miRNA mimic therapy in cancer. However, it remains unclear whether the clinical effects (both toxicity and anti-tumour activity) of MRX34 are related to specific gene-suppressing activity of the miR-34a nucleotide, a non-specific inflammatory effect of the double-stranded RNA (dsRNA) of the MRX34 formulation,^42–44^ or some other mechanism. Unravelling this biology will be critical for future development of this class of therapeutics.

Given the DEX pre-medication and that similar severe AEs did not occur with the same liposomal carrier for a different investigational oligonucleotide drug,^29^ it is unlikely that the severe toxicities of MRX34 were related to the liposome carrier.^14^ Both the apparently immune-related toxicities and the responses that followed an atypical pattern sometimes seen with other immune-activating agents, such as the CTLA-4 and PD-1/L1 immune checkpoint inhibitors,^45,46^ suggest an immune-mediated mechanism for the clinical effects of MRX34. The three responses reported in this study were observed in cancer types that have been previously shown to respond to immunotherapy.^47–49^ Whether the genes involved in tumour immune evasion, including PD-L1, that have been identified as targets for miR-34a down-regulation play a role in this mechanism remains to be determined.^19–21^ Two of the three responses occurred after patients were withdrawn from treatment (MRX34 and DEX), following potential pseudo-progression in one, raising the possibility that high-dose DEX pre-medication might suppress immune activation. This also remains an important question to be answered. Lower doses of first-cycle DEX (2 or 4 mg BID) pre-medication were tried in a number of patients, but this only resulted in increased toxicity, requiring a return to DEX 10 mg BID.

The pre-clinical AE profile derived from good laboratory practice toxicology studies performed with MRX34 in animals, including non-human primates (NHPs), did not predict the immune activation profile in humans. The main dose-dependent effects observed in these studies were decreased platelets, complement activation, and increased organ weights secondary to macrophage hypertrophy. Complement activation was not considered clinically significant, as it was not associated in monkeys with haemodynamic alterations or with formation of the biologically active split product C5a, which is a characteristic downstream manifestation of intensive complement activation.

This and a previous MRX34 paper using the initial BIW schedule summarise the first clinical experience with a new class of RNA-based drugs in oncology. Importantly, our study revealed the need to anticipate toxic effects from this class of drugs, specifically immune-mediated events, that may not be seen in pre-clinical toxicology models, including in NHPs. Effective delivery of these RNA constructs also remains an unresolved challenge, and the next generation of molecules will require an improved method of delivery to the tumour, with or without chemical modification of the construct, while avoiding systemic immune activation. Further exploration of various dosing schedules and pre-medication regimens is also needed. No additional clinical studies of MRX34 monotherapy or supportive translational research are being conducted or are planned at this time. In conclusion, while pharmacodynamics results demonstrate a proof-of-concept for miRNA-based therapeutics in cancer, this class of drugs requires further development to avoid immune-related toxicity in humans.

### Supplementary information

Supplemental Material for MRX34 in advanced solid tumors

## Data Availability

The datasets generated and/or analysed during the current study are not publicly available due proprietary restrictions but are available from the corresponding author on reasonable request.
