# Supplementary material for: Phase 1 study of MRX34, a liposomal miR-34a mimic, in patients with advanced solid tumours
Source: Br J Cancer. 2020 Apr 2;122(11):1630–7. doi: 10.1038/s41416-020-0802-1 (PMC7251107; doi:10.1038/s41416-020-0802-1)

Table S1. Summary of Pharmacokinetic Parameters by Dosing Cohort

Dose, mg/m^2	Day	n	Mean (±SD)				
			**AUC$_{last}$, h*ng/ml**	**Cl, ml/h/m^2**	**Cmax, ng/ml**	**T$_{½}$, h**	**Vd, L/m^2**
50	1	3	50835 (33280)	922 (859)	8775 (5589)	22.7 (14.1)	20.3 (10.3)
	5	4	819290 (365251)	76 (43)	16842 (6665)	53.6 (13.8)	5.2 (1.3)
70	1	25	62657 (37758)	1381 (1386)	10014 (4824)	14.2 (8.5)	22.0 (20.2)
	5	21	540240 (454298)	233 (187)	16800 (9737)	37.5 (11.5)	11.3 (8.4)
93	1	29	93099 (58955)	1179 (1342)	17608 (23459)	15.4 (13.0)	20.2 (23.6)
	5	20	584022 (357475)	204 (144)	23625 (19176)	39.2 (12.5)	9.9 (4.4)
110	1	9	135654 (54089)	655 (189)	20749 (6688)	12.6 (3.4)	12.0 (5.5)
	5	10	962932 (901408)	214 (191)	26204 (17499)	39.1 (14.8)	9.4 (5.3)

AUC$_{last}$, trapezoidal area under the concentration time curve from zero to the last measured concentration; Cl, clearance, Cmax, peak maximum blood concentration; T$_{½}$, half-life; Vd, volume of distribution.

Figure S1. MRX34 composition. MRX34 is a 23-nucleotide long, double-stranded, synthetic version of the microRNA miR-34a (a miR-34a “mimic”), encapsulated in a liposomal nanoparticle with a diameter of ~110 nm. The liposomal component contains amphoteric lipids that are cationic during liposome formation under acidic conditions to ensure efficient encapsulation of the negatively charged miR-34a mimic, and anionic *in vivo* at neutral pH to minimize immune stimulation, particle aggregation, and electrostatic adherence to the cellular membranes of endothelial cells.

Figure S1.

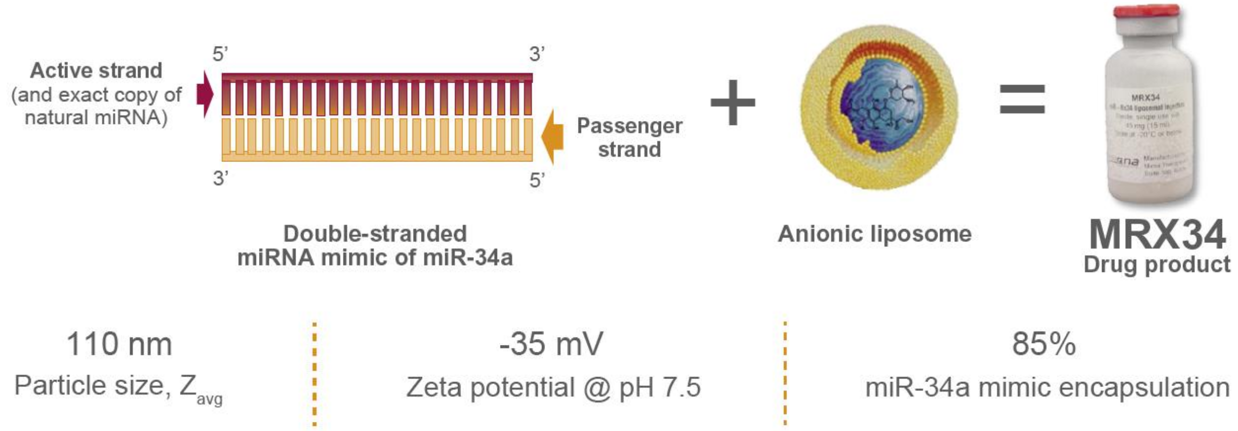

Figure S2. Blood concentration vs time profiles showing mean miR-34a mimic concentrations (ng/ml) at each dosing level during and following five daily infusions of MRX34 in cycle 1. Blood samples were collected prior to each infusion and at selected times post-infusion on days 1–5 and then once daily on days 6, 7, 8, and 14. Each data point reflects analysis of blood samples from at least three patients.

Figure S2.

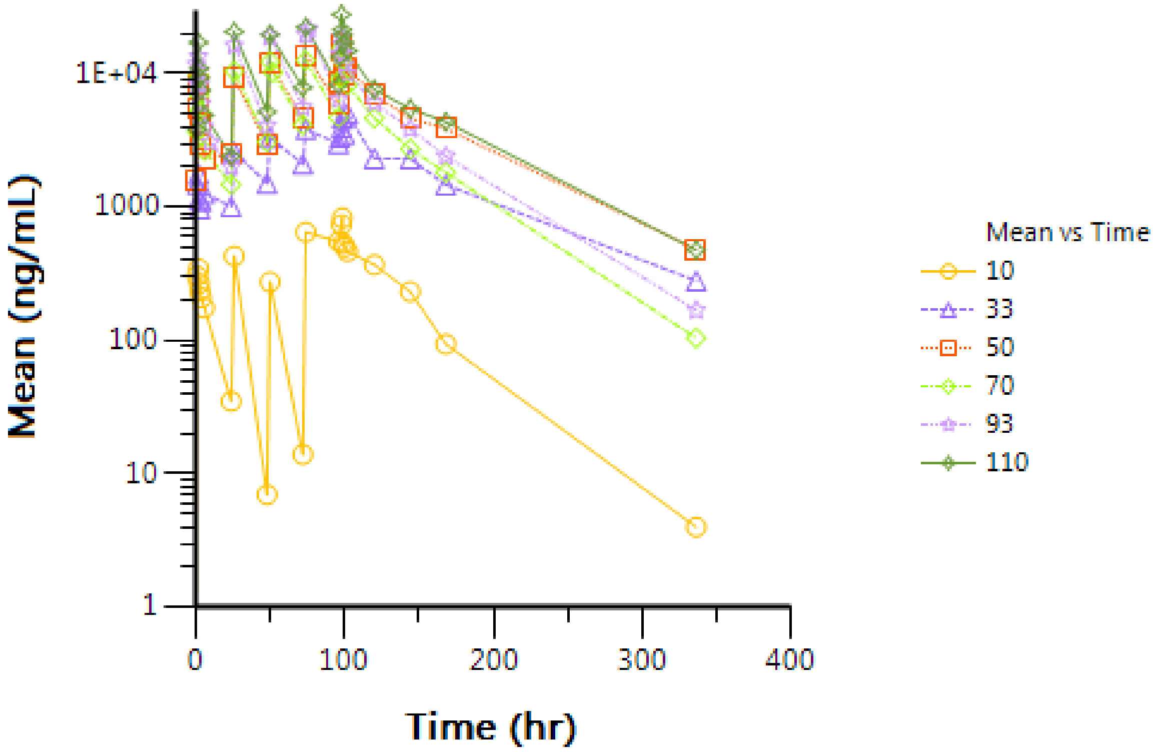

Supplement: Supplementary file 1 — Supplemental Material for MRX34 in advanced solid tumors [file 41416_2020_802_MOESM1_ESM.pdf]
